# Role of mucosal chemokines in the development of tissue-resident CD4^+^ and CD8^+^ T_RM_ cells to fend off herpes simplex infections

**DOI:** 10.3389/fimmu.2026.1661640

**Published:** 2026-02-19

**Authors:** Yassir Lekbach, Aziz A. Chentoufi, Swayam Prakash, Sweta Karan, Afshana Quadiri, Kathy K. Hormi-Carver, Joshua Christian Dorotta, Lbachir BenMohamed

**Affiliations:** 1 Laboratory of Cellular and Molecular Immunology, Gavin Herbert Eye Institute, School of Medicine, University of California, Irvine, Irvine, CA, United States; 2 Institute for Immunology, School of Medicine, University of California, Irvine, Irvine, CA, United States; 3 Department of Vaccines and Immunotherapies, TechImmune, LLC, University Lab Partners, Irvine, CA, United States

**Keywords:** CCL25, CCL28, cellular immunity, chemokines, CXCL14, CXCL17, memory CD4+ and CD8+ T cells, tissue-resident

## Abstract

The mucosal immune system represents the largest and most significant component of the immune network, providing critical defense against infectious pathogens at mucosal surfaces. Mucosal surfaces include the oronasal cavities, ocular surface, gastrointestinal tract, respiratory tract, and reproductive tract. Mucosal tissue-resident memory (T_RM_) CD4^+^ and CD8^+^ T_RM_ cells serve as sentinels and critical mediators of adaptive mucosal immunity, continuously trafficking to mucosal tissues to surveil and clear invading pathogens. The development of mucosal CD4^+^ and CD8^+^ T_RM_ cells is regulated by mechanisms distinct from those governing circulating effector memory (T_EM_) and central memory (T_CM_) T cells. Current models suggest that the generation, retention, and expansion of CD4^+^ and CD8^+^ T_RM_ cells within mucosal tissues are coordinated by mucosa-specific chemokines and adhesion molecules, thereby facilitating their selective homing and retention at mucosal surfaces. Among the 48 known chemokines, CXCL17, CCL25, CCL28, and CXCL14 have emerged as major key mucosal-specific chemokines that orchestrate mucosal CD4^+^ and CD8^+^ T_RM_ cell responses. This review (1) describes the roles of these four major mucosal chemokines in shaping T_RM_ cell-mediated immunity against mucosal pathogens, with a focus on herpes simplex virus type 1 (HSV-1) and type 2 (HSV-2), two infectious pathogens of the ocular and genital mucosae and (2) discuss harnessing these mucosal chemokine–receptor axes to develop a tissue-targeted Prime/Pull/Keep (PPK) herpes vaccine and immunotherapeutic strategies.

## 1 Introduction

Mucosal tissues serve as critical frontline barriers, continually exposed to a wide array of infectious pathogens (1). The mucosal immune system, the most significant component of the overall immune network, safeguards these vulnerable sites, including the oral, nasal, ocular, gastrointestinal, respiratory, and female reproductive tracts, the principal portals of pathogen entry (2).

It is now well established that a distinct subset of non-recirculating tissue-resident memory T cells (T_RM_) arises and persists within peripheral non-lymphoid mucosal tissues (3). These mucosal T_RM_ cells are phenotypically and functionally distinct from conventional circulating memory T cells, including effector memory (T_EM_) and central memory (T_CM_) subsets, which transit through the bloodstream and secondary lymphoid and non-lymphoid tissues (4–6). The developmental pathways regulating the generation, retention, and expansion of CD4^+^ and CD8^+^ T_RM_ cells within mucosal tissues are mechanistically distinct from those that guide conventional T_EM_ and TCM populations (7–10).

Current paradigms suggest that the tissue-specific trafficking of memory and effector CD4^+^ and CD8^+^ T cells to mucosal sites is governed by the coordinated expression of adhesion molecules and epithelial-derived chemokines, often in a tissue-restricted manner (11–19) (**Table 1**). The identification of epithelial-expressed chemokines, such as CCL25 in the small intestine and CCL28 in the respiratory and genital tracts, underscores the pivotal role of chemokines in directing T cell localization at mucosal surfaces under both homeostatic and inflammatory conditions (20, 35–39). This chemokine-mediated trafficking, known as lymphocyte homing, enables the selective accumulation of distinct T_RM_ subsets within mucosal tissues. Thus, chemokine-driven positioning of both antigen-presenting cells (APCs) and T cells is fundamental to the development of T_RM_-mediated immune surveillance and protection against mucosal infections.

**Table 1:** Timeline highlighting key advances in the study of chemokines CXCL17, CXCL14, CCL28, and CCL25, along with their receptors.

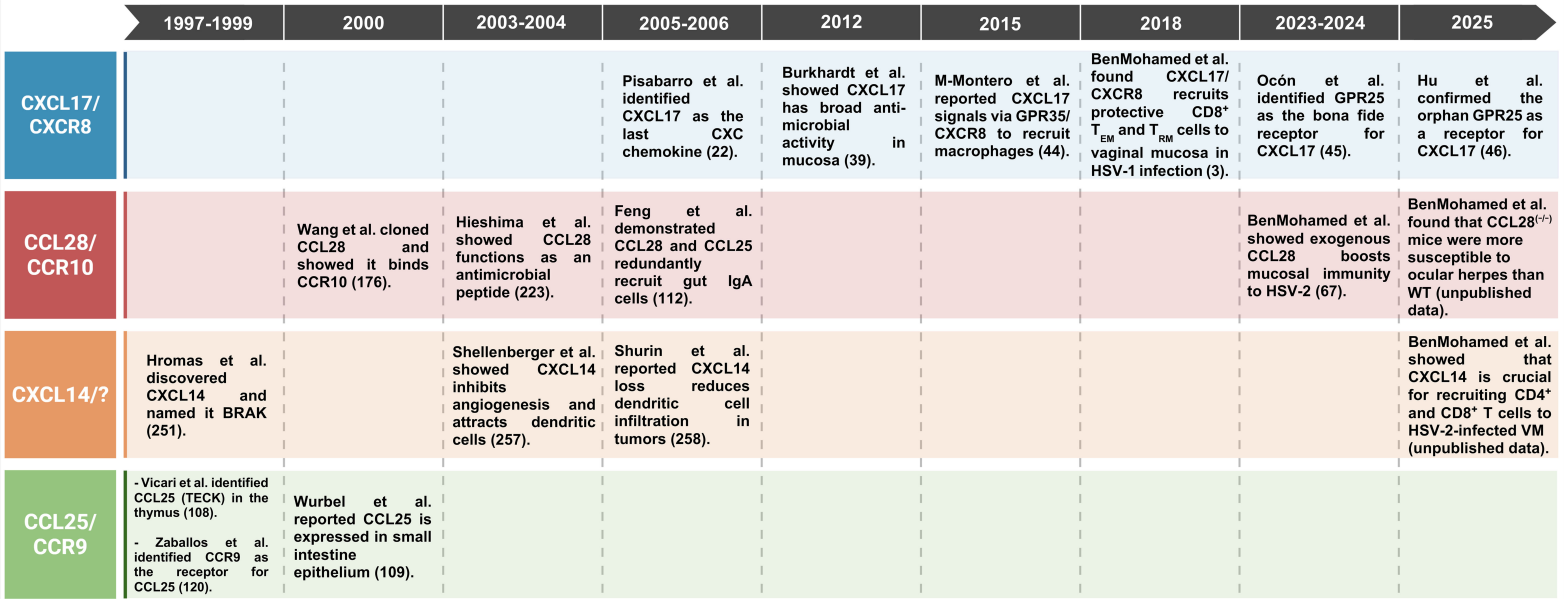

Chemokines constitute a family of approximately 50 small, secreted polypeptides characterized by chemotactic activity (40, 41) and classified into four subfamilies (C, CC, CXC, and CX3C) based on conserved cysteine motifs (42). Inflammatory chemokines predominantly regulate immune responses during infection and injury, whereas homeostatic chemokines orchestrate immune cell migration during tissue maintenance and immune homeostasis. By binding to G protein-coupled receptors on leukocytes, chemokines direct the trafficking of various immune cell subsets, including CD4^+^ and CD8^+^ T cells, across both lymphoid and non-lymphoid compartments (40, 43, 44). While several chemokines, such as CCL5, CXCL9, CXCL10, CXCL11, and CCL18, have well-characterized roles in non-mucosal T-cell recruitment (45–49), mucosa-associated chemokines, including CXCL17, CCL25, CCL28, and CXCL14, have emerged as key regulators of T-cell immunity at mucosal surfaces under both steady-state and inflammatory conditions. However, the specific mechanisms by which these epithelial-derived chemokines influence the development, localization, and maintenance of mucosal T_RM_ populations remain incompletely understood and are likely distinct from those governing conventional T_EM_ and T_CM_ subsets.

The T_RM_ cells reside permanently within non-lymphoid tissues, such as mucosal surfaces, and do not recirculate through the blood. They are identified by markers such as CD69 and CD103, which facilitate tissue retention and residence. T_RM_ cells serve as frontline sentinels, capable of rapid responses to local antigen re-exposure, thereby providing immediate protective immunity and reinforcing barrier defense (50–52). (**2**) The T_CM_ cells efficiently circulate between the blood and secondary lymphoid organs (SLOs), such as lymph nodes and spleen. They are characterized by expression of CCR7 and CD62L, which support homing to SLOs. Upon antigen challenge, TCM cells proliferate and give rise to effector T cells, bolstering the immune response throughout the body (21); and (**3**) The T_EM_ cells circulate through non-lymphoid tissues (NLTs) and blood, lacking lymphoid homing markers (CCR7, CD62L). T_EM_ cells are poised for rapid effector function and can respond promptly to pathogens in peripheral tissues, serving a surveillance role across different organ systems (53). Chemokines play a critical role in the development and localization of the T cell memory subsets described above (54). Chemokines such as CXCL9, CXCL10, and CCL25 promote the recruitment, retention, and compartmentalization of T_RM_ cells within barrier tissues by inducing the expression of specific homing and retention molecules (e.g., CD69, CD103). These chemokine-mediated signals help guide memory precursor T cells to mucosal sites where they can differentiate into T_RM_ cells and sustain long-term local immunity. Additionally, chemokines regulate the migration patterns of T_CM_ and T_EM_ cell subsets, ensuring efficient immune surveillance and the establishment of robust immunological memory at mucosal barriers (54).”

**Figure 1:**
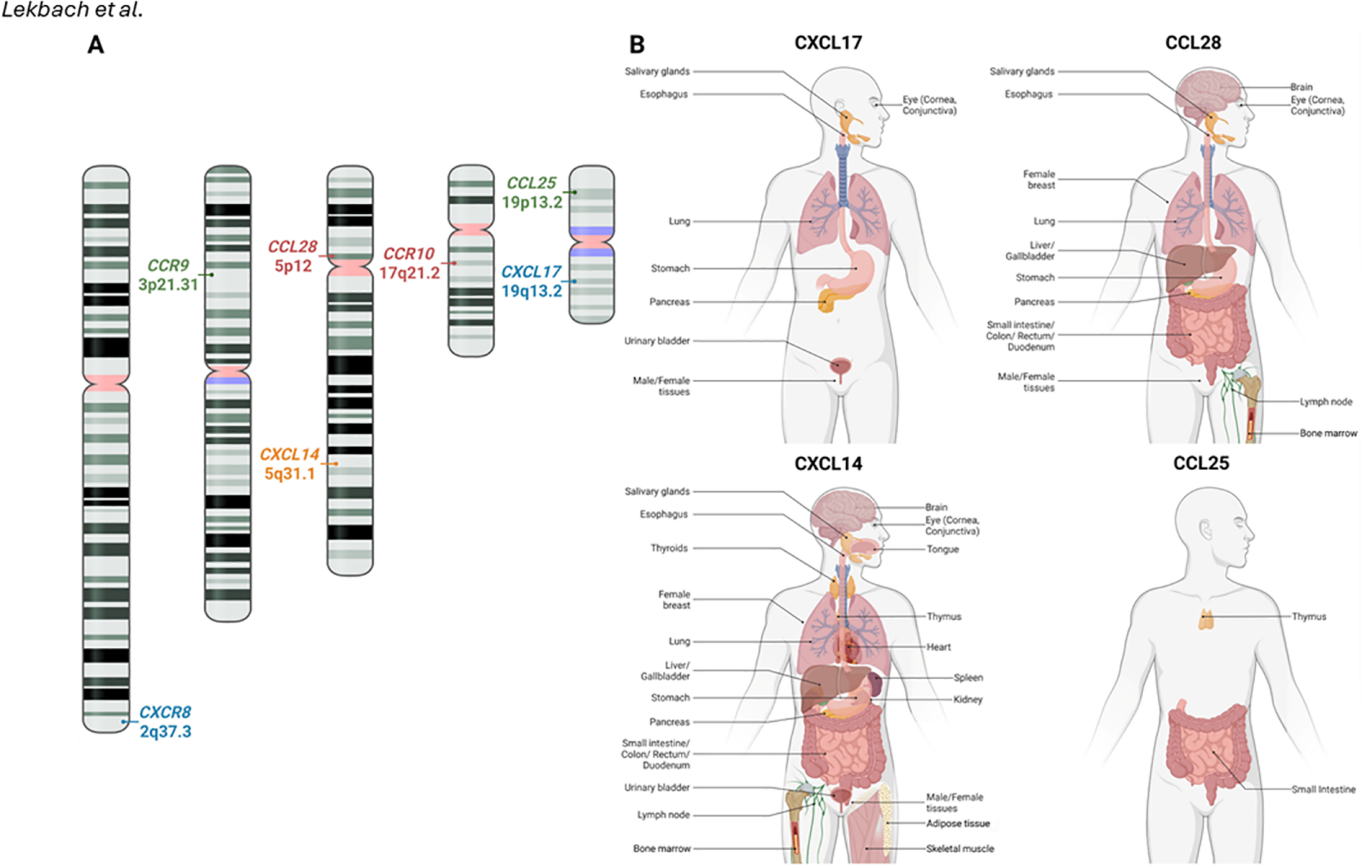
Mucosal chemokines chromosomal location and tissue expression in humans: **(A)** The chromosomal positions of chemokine genes CXCL17, CCL28, CXCL14, and CCL25, along with their respective receptors, are illustrated on human chromosomes. **(B)** The expression of CXCL17, CCL28, CXCL14, and CCL25 in human tissues. Data was retrieved from the Human Protein Atlas (www.proteinatlas.org). The receptor for CXCL14 has yet to be identified and created with BioRender.com.

**Figure 2:**
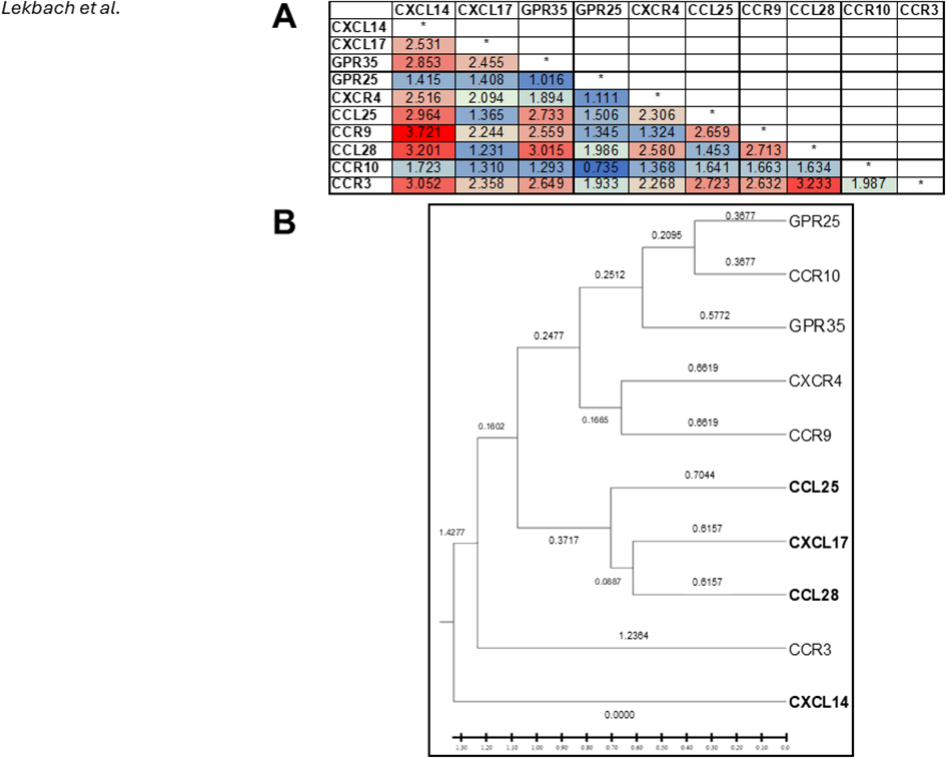
Evolutionary comparison between different mucosal chemokines and their receptors: **(A)** Genetic distances based on Maximum Composite Likelihood model among the mucosal chemokines (CXCL14, CXCL17, CCL25, CCL28) and their receptors (GPR35, GPR25, CXCR4, CCR9, CCR10, and CCR3). Nucleotide sequences were specific to human for these mucosal chemokines and their receptors. In comparison to CXCL14, results indicate less genetic distance (indicating genetic similarity) among other mucosal chemokines CCL28 (3.201), CXCL17 (2.531), and CCL25 (2.964) respectively. **(B)** Phylogenetic analysis performed to evaluate evolutionary relationship among the mucosal chemokines and their receptors using the UPGMA method. The optimal tree is shown. The tree is drawn to scale, with branch lengths (next to the branches) in the same units as those of the evolutionary distances used to infer the phylogenetic tree. The evolutionary distances were computed using the Maximum Composite Likelihood method and are in the units of the number of base substitutions per site. All ambiguous positions were removed for each sequence pair (pairwise deletion). There was a total of 56106 positions in the final dataset for the nucleotide sequences of the 10 mucosal chemokine ligands and receptors. Evolutionary analyses were conducted in MEGA11.

In this review, we focus on the physiological roles of the major mucosal-specific chemokines CXCL17, CCL25, CCL28, and CXCL14 in shaping CD4^+^ and CD8^+^ T_RM_ cell-mediated immunity to mucosal infectious diseases (**Figure 1** and **Table 1**). The phylogenetic analysis of all these mucosal chemokines (CXCL14, CXCL17, CCL25, CCL28) and their receptors (GPR35, GPR25, CXCR4, CCR9, CCR10, and CCR3) revealed, notably, a smaller genetic distance among the mucosal chemokines, indicating an evolutionary relationship among them (**Figure 2**).

Furthermore, we discuss emerging strategies targeting chemokine–receptor axes to enhance mucosal vaccine efficacy and develop novel immunotherapeutic approaches against HSV-1 and HSV-2 infections.

## 2 The mucosal chemokine (C-X-C motif) ligand 17

### 2.1 *The* CXCL17 *chemokine is primarily expressed by* mucosal tissues of the oral cavity, gastrointestinal, respiratory, and genital tracts

The mucosal chemokine CXCL17 was first characterized in 2006 by Pisabarro et al. (Genentech Inc., San Francisco, CA) as a monocyte-attracting chemokine (**Table 1**) (37). CXCL17 is also referred to as Vascular Endothelial Correlated Chemokine (VCC-1) or VEGF Co-regulated Chemokine 1, and as Dendritic Cell and Monocyte Chemokine-like Protein (DMC) (37, 38). The CXCL17 gene is located on chromosome 19 at position 19q13.2 (**Figure 1A**). It consists of four exons that encode a 119-amino-acid propeptide, with an approximate molecular mass of ~13.8 kDa in humans and ~13.6 kDa in mice (37, 38). CXCL17 is constitutively expressed in the oral mucosal tissues, as well as the gastrointestinal, respiratory, and genital tracts (**Figure 1B**) (22, 55, 56). Additionally, CXCL17 has been implicated in regulating infection and inflammation in the gastrointestinal, respiratory, and female reproductive systems (23). Notably, it exhibits broad antimicrobial activity at mucosal surfaces (24). Nevertheless, the precise mechanisms by which CXCL17 mediates mucosal protection against invading pathogens remain largely unclear.

In 2022, Shabgah et al. reviewed the role of the chemokine CXCL17 in infection and inflammation (23). CXCL17 plays a crucial role in maintaining homeostasis across various mucosal tissues by regulating myeloid cell recruitment, promoting angiogenesis, and controlling microbial populations (23). Their findings confirmed that CXCL17: (1) under homeostatic conditions, exhibits anti-inflammatory, antibacterial, and chemotactic activities; (2) under pathological conditions such as malignancies, promotes angiogenesis, metastasis and cellular proliferation; (3) possesses anti-tumor properties; (3) is implicated in the pathogenesis of diseases including idiopathic pulmonary fibrosis, multiple sclerosis, asthma, and systemic sclerosis; and (5) may serve as a biomarker for disease diagnosis and prognosis via its dysregulation.

In 2024, Dominguez-Lopez et al. reported significant upregulation of CXCL17 expression in conjunctival and corneal mucosal epithelial cells in patients with dry eye disease (57). These findings suggest that corneal and conjunctival epithelial cells within the ocular mucosal immune system (OMIS) may serve as a source of CXCL17 during ocular infections and inflammatory responses (57–59).

### 2.2 CXCR8 (GPR35), GPR25, and CXCR4 are receptors of CXCL17 chemokine

Back in 2010, Fallarini et al. were the first to report on the expression of the orphan G protein-coupled receptor (GPR35) on human iNKT cells (60). Later, in 2015, Maravillas-Montero et al. confirmed that CXCL17 signals through GPR35, which was later renamed CXCR8 (61). Several studies have suggested that CXCR8 may have both pro-inflammatory and anti-inflammatory effects (60, 61). More recently, GPR25 has been identified as a receptor for CXCL17 by multiple groups (62, 63).

In 2018, Pease et al. reported the existence of another CXCL17 receptor, CXCR4 (20). Similarly, in 2018, Hill et al. confirmed that CXCR4 is the other receptor for CXCL17, in addition to CXCR8 (20, 35, 64). In 2023, Peace et al. demonstrated that the C-terminal fragments of CXCL17 readily bound glycosaminoglycans (GAGs) and may serve as prototypic inhibitors of CXCR4 function (20, 41, 65). Later, in 2024, Hill et al. confirmed that CXCL17 was an allosteric endogenous inhibitor of CXCR4, an effect that was mimicked by GAGs, surfen, and protamine sulfate (20, 35, 64). Disruption of putative GAG binding domains in CXCL17 prevented CXCR4 binding (20, 35, 64).

A characteristic feature of chemokines is their similar three-dimensional structure, four conserved cysteines that form two disulfide bonds, and binding to glycosaminoglycans (GAGs). Both disulfides are essential for receptor activation, and GAG binding is critical for the directed migration of leukocytes. However, CXCL17 has only three conserved cysteines (instead of four) and thus may not adopt the typical chemokine structure. It also exhibits a distinct distribution of positively charged residues, suggesting a novel GAG-binding mechanism. Another unusual feature of CXCL17 is that it is active only at high concentrations, with optimal activity at concentrations up to 1000-fold higher than those typically observed for most chemokines (µM vs. nM). Below, we review and discuss how CXCL17 regulates the homing of neutrophils, monocytes/macrophages, dendritic cells, and T cells to mucosal tissues (20, 35–39).

### 2.3 CXCL17 is a pro-inflammatory chemokine that promotes early neutrophil recruitment to inflamed mucosal tissues

Identifying the underlying mechanism of CXCL17’s activity in homeostatic, inflammatory, and pathological situations holds promise for the development of novel treatment strategies. CXCL17 has been implicated in several human pathologies and inflammatory diseases (39, 41, 56, 66–68). More recently, in 2024, Lowry et al. demonstrated that, compared to their littermate control wild-type (WT) mice, CXCL17^(-/-)^ deficient mice were significantly impaired in peritoneal neutrophil recruitment following lipopolysaccharide (LPS) challenge (69). The CXCL17^(-/-)^ deficient mice showed: (1) dysregulated levels of inflammatory mediators CXCL1, CXCR2, and IL6, all of which directly impact neutrophil recruitment (69); and (2) no difference in monocyte recruitment following LPS-challenge (69). The study confirmed that the CXCL17 pro-inflammatory chemokine plays a crucial role in early inflammatory responses by promoting neutrophil recruitment to the lungs (69).

### 2.4 CXCL17 promotes the recruitment of dendritic cells and macrophages to inflamed mucosal tissues of the respiratory tract

In 2014, Burkhardt et al. demonstrated in a mouse model that CXCL17 is a central chemotactic mediator for macrophage recruitment into the lungs (70). Compared with WT mice, Cxcl17^(-/-)^mice displayed a significant reduction in the frequency of macrophages in their lungs (70). In addition, the authors detected a concurrent increase in a new population of macrophage-like cells in the lungs of CXCL17^(-/-)^ deficient mice, characterized by an F4/80^(+)^CD11c^(mid)^ phenotype (70). Later, in 2019, Hernandez-Ruiz et al. confirmed that CXCL17^(-/-)^ deficient mice displayed lower frequencies of macrophages and dendritic cells in the lungs. In 2021, Choreño-Parra et al. reported that CXCL17 production increased in the lungs of *mice infected with M. tuberculosis*, and that mice treated with recombinant CXCL17 showed increased accumulation of lung myeloid cells (71). Taken together, these results suggest that CXCL17 is a chemoattractant for both macrophages and dendritic cells in mucosal tissues of the respiratory tract.

**Figure 3:**
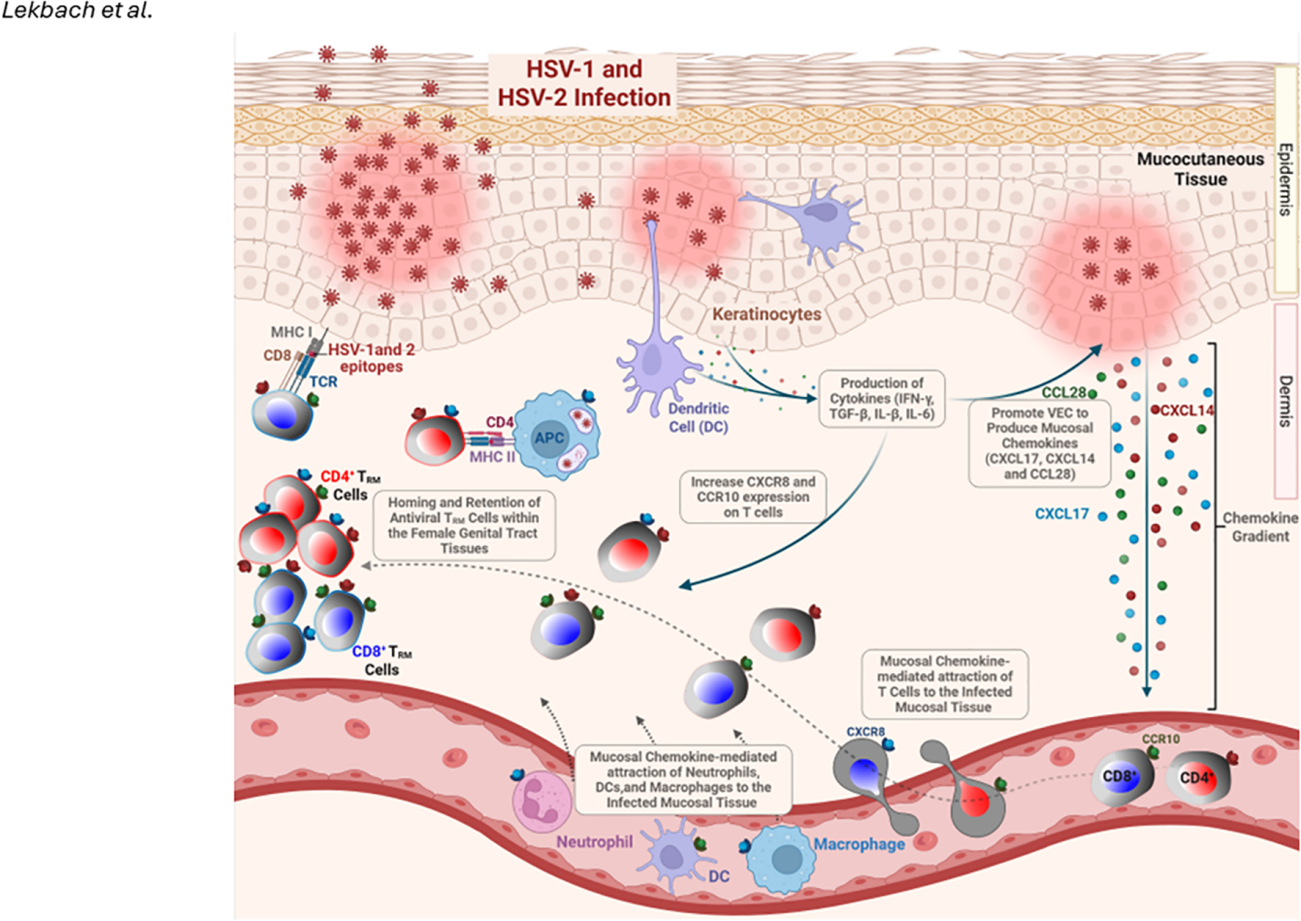
Role of Mucosal Chemokines in T Cell Homing and Retention in the Mucocutaneous Tissue During HSV-1/2 Infection: HSV-1/2 infection of vaginal epithelial cells (VECs) and keratinocytes triggers the production of inflammatory cytokines (IFN-γ, TGF-β, IL-1β, IL-6), which promote them to: (1) produce mucosal chemokines CXCL17, CXCL14, and CCL28, and (2) upregulate CXCR8 and CCR10 expression on T cells. These chemokines facilitate the homing and retention of antiviral tissue-resident memory (T_RM_) cells in the genital tract while also recruiting dendritic cells, neutrophils, and macrophages to the site of infection. Created with BioRender.com.

Since CXCL17 facilitates the recruitment of both macrophages and dendritic cells, two primary antigen-presenting cells (APCs), into mucosal tissues (20, 35–39), we will review how CXCL17 regulates the trafficking and localization of CD4+ and CD8+ T cells to combat invading mucosal pathogens (**Figure 3**).

### 2.5 The CXCL17/CXCR8 chemokine/receptor pair regulates effector and regulatory CD4^+^ and CD8^+^ T cells trafficking and localization into inflamed and infected mucosal tissues

Studies have shown that GPR35 activation can stimulate the production of pro-inflammatory cytokines and facilitate the movement of immune cells, including effector CD4^+^ and CD8^+^ T_RM_ cells, towards inflammatory and infected mucosal tissues of gastrointestinal tract, respiratory tract, and female genital tract (28, 60, 72–80). Conversely, other investigations have suggested that GPR35 possesses anti-inflammatory properties in mucosal tissues by inhibiting the generation of inflammatory mediators and promoting the differentiation of regulatory T cells (T_reg_ cells) (28, 60, 72–82).

Later, in 2018, we reported that CXCL17 is involved in the recruitment of CD8^+^ T cells that mediate protective immunity against sexually transmitted herpes infection (4, 83). We used herpes simplex virus type 1 (HSV-1) as a model of a genital viral pathogen. We selected HSV-1 because (*i*) it is widely present in more than 3.7 billion people worldwide and (*ii*) it is becoming an increasingly common cause of genital infection, mainly in developed countries (84). We found that intravaginal HSV-1 infection of WT B6 mice increased the frequency of HSV-specific IFN-γ-producing cytotoxic CXCR8^+^CD8^+^ T cells in the female genital tract, and that this recruitment was associated with protection against genital herpes infection and disease. In contrast, when compared to WT B6 mice, following HSV-1 infection, the female genital tract of CXCL17^(-/-)^ deficient mice developed significantly fewer HSV-specific functional IFN-gamma-producing cytotoxic CXCR8^+^CD8^+^ T_RM_ cells. It generates more exhausted VISTA^+^TIGIT^+^CD8^+^ T cells that fail to control genital herpes infection (4, 83). These observations strongly suggest that the CXCL17/CXCR8 axis is a major regulator of female genital tract-resident CD103^high^CD8^+^ T_RM_ cell responses, which lead to the clearance of genital herpes infection (4, 83). Whether the increase in the number of CXCR8^+^CD8^+^ T cells and CCR10^+^CD8^+^ T cells is due to increased recruitment, formation, retention, and/or expansion of these cells within the female genital tract remains to be determined.

In 2019, Hernandez-Ruiz et al. reported that CXCL17 is also a chemoattractant that suppresses myeloid cell chemoattraction and recruits regulatory T cells (85). Compared with WT littermate mice, CXCL17^(-/-)^ mice developed more severe disease in a T-cell-dependent model of experimental autoimmune encephalomyelitis (EAE) (85). After immunization with myelin oligodendrocyte peptide, only 44% of CXCL17^(-/-)^ deficient mice were still alive vs. 90% for WT mice. During EAE, CXCL17^(-/-)^ mice exhibited reduced myeloid cell infiltration into the CNS and higher serum levels of inflammatory cytokines (85). In 2021, Choreño-Parra et al. reported that, in humans, lower serum CXCL17 levels are observed among active pulmonary TB patients than among subjects with latent TB infection and healthy controls, suggesting a protective role (71).

These reports suggest that CXCL17 may regulate the development of protective mucosal CD4^+^ and CD8^+^ T_RM_ cells at steady state and during inflammatory and infectious conditions (**Figure 3**).

### 2.6 Role of CXCL17/CXCR8 chemokine/receptor axis in the development of genital tract mucosal CD4^+^ and CD8^+^ T_RM_ cells against genital herpes

The genital tract mucosal surface represents one entry route of herpes simplex virus type 1 and/or type 2 (HSV-1 and HSV-2) (86, 87). Herpes infection is widespread in human populations, with a higher incidence in women than in men (84, 88–91). HSV viruses cause initial infections in the mucocutaneous tissues of the mouth, lips, genital tract, nose, or eyes (92). Most HSV-seropositive women are asymptomatic (ASYMP) (93–96). They do not experience any recurrent herpetic genital disease even though the virus spontaneously reactivates from latency and sheds multiple times each year in their vaginal secretions (89, 90, 97, 98). In contrast, a small proportion of HSV-seropositive women are symptomatic (SYMP) and experience endless recurrences of herpetic disease, usually multiple times a year (86, 87), often requiring continuous antiviral therapy (i.e., acyclovir and derivatives).

Mucosa-resident memory T_RM_ cell subsets are phenotypically and functionally distinct from conventional T_EM_ and T_CM_ cell subsets that circulate through the blood to access lymphoid and non-lymphoid tissues (4–6, 99–101). The mechanisms that regulate the generation, retention, and expansion of genital tract mucosal CD4^+^ and CD8^+^ T_RM_ cells are distinct from those regulating conventional circulating T_EM_ and T_CM_ cells (7–10). Direct experiments in animal models (102–104) and indirect evidence in humans (105, 106) suggest that the induction of robust, polyfunctional T_RM_ cells residing within the vaginal submucosal tissues contributes to the successful control of herpes infection in the female genital tract (107). However, the female genital tract tissues appear to be immunologically restricted and remain “a closed immunological compartment, “ resistant to the homing of peripheral T cells that may originate from the draining lymph nodes and circulation (108, 109). Genital herpes infection of vaginal epithelial cells (VECs) likely triggered the production of inflammatory cytokines, such as IFN-gamma TGF-beta, IL-1beta, and IL-6, which promotes VECs: (1) to produce mucosal chemokines, such as CXCL17 and CCL28, and (2) increased CXCR8 expression on T cells contributing to homing and retention of antiviral T_RM_ cells within the female genital tract tissues (**Figure 3**) (108, 109).

Specifically, CD8^+^ T_RM_ cells mediate protection against genital herpes in both human and mouse models (90, 103, 110–114). CD8^+^ T_RM_ cells are maintained in the dermal-epidermal junction of the female genital tract (32, 115). We recently demonstrated the role of CXCL17, a mucosal chemokine, in the development of anti-herpes CD8^+^ T cell memory (T_RM_) cells within the female genital tract (4). We showed that following intravaginal HSV-1 infection of WT B6 mice, the CXCL17 production is strongly induced in the female genital tract, and CXCL17 local expression paralleled an increase in both the number and effector function (IFN-gamma-production and CD107^a/b^ degranulation) of HSV-specific CXCR8^+^CD8^+^ T_RM_ cells associated with protection against genital herpes infection and disease (4). In contrast, HSV-1 infection of CXCL17^(-/-)^ deficient mice resulted in an increased number of female genital tract-resident exhausted VISTA^+^TIGIT^+^CD8^+^ T_RM_ cells, associated with a failure to contain genital herpes infection and disease (4). These results were the first to suggest a crucial role of the CXCL17/CXCR8 chemokine axis in the development of protective female genital tract mucosal CD8^+^ T_RM_ cells (4). Besides effector CD8^+^ T cells, CD4^+^ T helper cells appeared to indirectly control the migration of CD8^+^ T cells through the secretion of IFN-gamma and local induction of chemokine secretion in the infected female genital tract tissue (34). We recently reported that the CXCL17 chemokine plays a role in CD4^+^ T cell immunity in the female genital tract (4, 83, 116). A recent study also showed that chemokines secreted by a local macrophage network maintained vaginal T_RM_ cells in memory lymphocyte clusters (MLCs) independently of circulating memory T cells and dendritic cells (51).

In 2018, we reported that CXCR8 is constitutively expressed in female genital tract-derived B cells, T cells, dendritic cells (DCs), and monocytes/macrophages (4). CXCR8 expression on these cells increased following HSV-2 genital herpes infection (4). Moreover, both the frequency of female genital tract-resident CXCR8^+^CD8^+^ T cells and the expression level of CXCR8 were significantly increased following the intravaginal administration of exogenous CXCL10 in HSV-2-infected mice (4). These results suggest that, although the CXCL10/CXCR3 axis contributes to the recruitment of CD8^+^ T cells into the female genital tract, their retention within the mucosal epithelium for an extended period is controlled by the CXCL17:CXCR8 axis (4).

Therapeutic manipulation of the immune system, referred to as immunotherapy, is an attractive strategy for addressing genital herpes disease, HSV-1 and HSV-2 shedding, and ultimately reducing HSV-1 and HSV-2 transmission in the community (93–96). For successful herpes immunotherapy to occur, one must first identify the immune mechanisms underlying the apparent protection observed in seropositive asymptomatic women, who appear to contain infection and disease immunologically. Understanding the mechanisms by which CD8^+^ T_RM_ cells develop, maintain, and expand in the female genital tract tissue will have a significant impact not only on genital herpes but also on other sexually transmitted infectious pathogens (STIs).

## 3 The mucosal chemokine (C-C motif) ligand 25 in human health and disease

### 3.1 *The* CCL25 *chemokine is primarily expressed by* mucosal tissues of the nasal cavity, gastrointestinal tract, and mammary glands

Herpes simplex virus (HSV) can infect the gastrointestinal tract, leading to conditions such as herpes gastritis, herpes proctitis, herpes esophagitis, and herpes colitis, which are rare but serious health concerns in immunocompromised patients (27, 117, 118). While the CCL25 mucosal chemokine is highly expressed in gut-associated lymphoid tissues (GALT), its role in gut immunity and immunopathology in response to herpes gastritis, herpes proctitis, herpes esophagitis, and herpes colitis remains to be fully determined.

The mucosal chemokine CCL25 belongs to the CC chemokine family, which is also known as TECK (Thymus-Expressed Chemokine). Vicari et al. first reported in 1997 that CCL25 is a thymic dendritic cell-specific CC chemokine involved in T-cell development (**Table 1**) (119). The CCL25 chemokine was first identified in the thymus of mice and mapped to chromosome 8. The primary source of CCL25 in the thymus was thymic dendritic cells and mucosal epithelial cells (**Figure 1B**) (120). In contrast, bone marrow-derived dendritic cells do not express CCL25. Later, in 1998, the human CCL25 gene (*SCYA25*) was mapped to chromosome 19 (**Figure 1A**) (121). Human CCL25 is produced as a 151-amino acid protein precursor (121). In humans, mice, and pigs, CCL25 plays a crucial role in the segregation and compartmentalization of the mucosal immune system by recruiting B and T cells to specific mucosal locations (121, 122). CCL25 attracts an overlapping subpopulation of IgA-secreting B cells, which are concentrated in the small intestine and its draining lymphoid tissues (33, 123–126).

Meurens et al. reported the cloning and the sequencing of ovine CCL25 and the subsequent assessment of its mRNA expression by quantitative PCR in several tissues, including thymus, gut-associated lymphoid tissue, and mammary gland, from young and adult sheep and in the fetal lamb during the development of the immune system (127–129). CCL25 mRNA is highly expressed in the thymus and gut. These results are consistent with observations in mice, humans, and other species (127–129). In fetuses, CCL25 mRNA is expressed early in the thymus, small intestine, and nasal mucosa. Furthermore, their expression increased towards the end of gestation (127–129). Consequently, CCL25 may play a crucial role in the lymphocyte colonization of fetal tissues, thereby facilitating the development of a functional immune system (127–129).

### 3.2 *The* CCL25 chemokine binds to *CCR9 receptor* expressed on specific *subsets of mucosal-resident* CD4^+^ and CD8^+^*T cells*

The CCR9 is a G protein-coupled receptor and the main receptor for CCL25 chemokine. Zaballos et al. first reported that the receptor for the CCL25 ligand is C-C chemokine receptor type 9 (CCR9) (130). In 2015, Kim et al. discovered CCR9-mediated signaling through beta-catenin and confirmed CCL25 as a CCR9 antagonist (131). The CCR9 receptor for CCL25 was later confirmed in 2000 by Gosling et al., who reported that it is expressed on dendritic cells (DCs) and T cells (132). CCR9 was also expressed on subsets of mucosal CD4^+^ and CD8^+^ T cells, gamma-delta T cells, plasmacytoid dendritic cells (pDCs), IgA plasma blasts, IgA plasma cells, and intraepithelial lymphocytes (IELs) from the mucosal tissues cited above (133, 134). CCR9 is specifically co-expressed on a subset of gut-homing B and T cells expressing the integrin α4β7, immunoglobulin A (IgA)- secreting B cells from the gastrointestinal tract. CCR9 drives the migration of these B and T cell subsets to gradients of its cognate ligand, the CCL25 chemokine (133, 134). CCR9 expression defines a subset of peripheral blood T cells with a mucosal phenotype and a Th_1_ or T-regulatory 1 cytokine profile (135–139). The CCR9 receptor equips these T cells to respond to CCL25 (140). Notably, CCR9 appears to be predominantly expressed on CD8^+^ T cells but less so on CD4^+^ T cell subsets, thereby imposing distinct tissue tropisms on CD4^+^ and CD8^+^ T cells in various mucosal tissues.

### 3.3 Role of CCL25/CCR9 chemokine/receptor axis in the development of mucosal tissues-resident CD4^+^ and CD8^+^ T cells

Naive CD4^+^ and CD8^+^ T cells require activation in lymphoid tissues before differentiating into effector or memory T cells capable of trafficking to nonlymphoid mucosal tissues (141, 142). Distinct non-recirculating T_RM_ cell subsets are developed and retained within peripheral non-lymphoid mucosal tissues (4–6, 99–101, 143). These mucosa-resident T_RM_ cells are phenotypically and functionally distinct from conventional memory T cells, which are defined as T_EM_ and T_CM_ cells that circulate in the blood and access lymphoid and non-lymphoid tissues (4–6, 99–101, 143). The mechanisms that regulate the generation, retention, and expansion of mucosal CD4^+^ and CD8^+^ T_RM_ cells within the mucosal tissues are distinct from those regulating conventional circulating T_EM_ and T_CM_ cells (7–10). While there are well-established T-cell-attracting chemokines (i.e., CCL5, CXCL9, CXCL10, CXCL11, and CCL18) (45–49), several mucosal chemokines also play a role in shaping mucosal T cell immunity (92, 144–159). Tissue-selective trafficking of memory and effector CD4^+^ and CD8^+^ T cells is mediated by distinct combinations of adhesion molecules and chemokines (20, 35–39). The role of CCR9-CCL25 in immune cell migration, including effector and regulatory T cells, is well studied in both homeostasis and disease (133).

#### 3.3.1 Role of the CCL25/CCR9 axis in the development of gut-resident T cells

The discovery of the related epithelial-expressed CCL25 chemokine in the small intestine and other diverse mucosal sites highlights an essential role for CCL25 in controlling memory and effector CD4^+^ and CD8^+^ T cells trafficking and localization in the intestine. Constitutively expressed epithelial chemokines may help determine the character of local T-cell responses and contribute to the organization of the gut immune system. CD4^+^ and CD8^+^ T cells primed by intestinal dendritic cells (DC) express the gut-homing receptors CCR9 and alpha4beta7 (α4β7), which recognize CCL25 and mucosal address cell-adhesion molecule-1 in the intestine, promoting the development of regional immunity (160–165).

The role of CCL25, a mucosal chemokine, in trafficking T cells into the gastrointestinal tract and shaping intestinal immunity was reported in 2002 by Kunkel, Campbell, and Butcher (166). Staton et al. later reported, in 2004 and 2006, that CD8^+^ recent thymic emigrants (RTEs) migrated directly into the small intestine and that CCR9, CCL25, and α4β7 integrin were all required for gut entry of these CD8^+^ RTEs (141, 142). After antigen-driven T cell receptor stimulation, the intestinal CD8^+^ RTEs proliferated and acquired a specific surface phenotype resembling that of intraepithelial T cells. These CD8^+^ RTEs efficiently populated the gut of lymphotoxin-alpha-deficient mice, which lack lymphoid organs (141, 142). These studies challenged the traditional concept of naive CD4^+^ and CD8^+^ T cell trafficking, suggesting that RTEs may play a role in maintaining a specific and diverse CD4^+^ and CD8^+^ T cell repertoire at mucosal surfaces (141, 142).

CCR9 overexpression was detrimental to the proper tissue distribution and terminal differentiation of CD4^+^ T cells in the gut epithelium (140). A recent study demonstrated that the expression of CCR9 interferes with tissue trafficking and differentiation of CD4^+^ T cells in the small intestine intraepithelial tissues (140). Specifically, the differentiation of small intestine epithelial-resident CD4^+^ T cells into immunoregulatory CD4^+^CD8αα^+^ T cells was impaired by the overexpression of CCR9 and conversely increased by the genetic deletion of CCR9 (140). The study revealed a previously unappreciated role for CCR9 in the tissue homeostasis and effector function of CD4^+^ T cells in the gut (140). More recently, in 2022, Li et al. reported that the chemokine receptor CCR9 suppresses the differentiation of CD4^+^CD8 ^α (+)^ intraepithelial T cells in the gut (140). In a mouse model, the recombinant CCL25 protein exhibited chemotactic activity for activated macrophages, dendritic cells, and thymocytes (140). CCR9 ameliorates inflammation in a T cell-mediated mouse colitis model (122).

Pathak et al. also demonstrated the role of the CCL25/CCR9 chemokine ligand/receptor pair in mucosal APC and T cell differentiation (133). Using a dextran sodium sulfate (DSS)-induced gut inflammation model, they demonstrated that CCR9^(+)^ dendritic cells (DCs), specifically CD11b^(-)^ CD103^(+)^ DCs, were significantly increased in the GALT compared to control WT mice (133). These CCR9^(+)^ DCs express lower levels of MHC II and CD86 molecules and possess regulatory surface markers, including FasL and latency-associated peptide (LAP), in the GALT. CCR9 signaling in DCs drives the differentiation of Foxp3+ T_regs_ and suppresses the allergic IgE response in the gut (133, 134). In the presence of CCL25, CCR9^(+)^ DCs promoted *in vitro* differentiation of Foxp3^(+)^ regulatory CD4^(+)^ T cells (T_regs_) (133). CCL25-induced differentiation of T_regs_ was due to intrinsic signaling in the DCs but not through CD4^(+)^ T cells, which was driven by the production of thymic stromal lymphopoietin (TSLP) rather than IL-10 (133). Furthermore, the adoptive transfer of CCR9^(+)^ DCs in WT C57BL/6 mice promoted T_regs_ and reduced Th17 cells in the GALT, thereby suppressing the OVA-specific gut-allergic response (133).

A 2022 study in a zebrafish model reported that CCR9^+^ T cells are recruited to a band in the lamina propria next to the muscular mucosa in which frequent CCL25-expressing cells are present. CCR9^+^ T cells interact with APCs for several minutes in a process mediated by Connexin 43 (167). This type of interaction was observed in both homeostasis and inflammation states, with the interaction being longer and more frequent during inflammation (167). The study suggested that the mucosal immune response in the intestinal mucosa is organized and organized into specific regions with specialized microenvironments and functions (167).

#### 3.3.2 Role of CCL25 chemokine in the development of genital and gastrointestinal tract-resident protective CCR9^+^β7^+^ T cells

The location and manner of antigen-presenting cells (APCs) and T cells within the mucosal tissue are essential in the defense against mucosal infectious pathogens.

In 2008, Reinhart et al. measured chemokine and cytokine mRNA levels in multiple lymphoid tissue compartments from SIV-infected vs. uninfected cynomolgus macaques (*Macaca fascicularis*) (168). It was found that CCL25 mRNA levels in SIV-infected lymphoid tissues were decreased, and that CCL25 levels were negatively correlated with the numbers of proliferating and apoptotic cells (168). *In vitro* analyses revealed that CCL25 could reduce SIV-induced apoptosis (168). These findings suggest that increased apoptosis in lymphoid tissues, resulting from reduced levels of the anti-apoptotic chemokine CCL25, may contribute to the loss of immune function following pathogenic SIV infection (168).

In 2011, Cromwell et al. reported that SIV-specific CD8^+^ T cells are enriched in the female genital tract of rhesus macaques and express receptors for inflammatory chemokines, including CXCR3, CCR5, and CCR9 (169). Cromwell et al. found that the frequency of SIV-specific CD8^+^ T cells was 3- to 30-fold higher in genital mucosal tissues compared to peripheral blood (169). SIV-specific CD8^+^ T cells in genital mucosal tissues expressed high levels of CXCR3 and CCR5, chemokine receptors commonly expressed on memory T cells that home to inflamed tissues (169). Cells expressing CXCR3 colocalized with its chemokine ligand CXCL9 in the vaginal lamina propria (169).

In 2012, Mavigner et al. investigated the trafficking of CD4^+^ T cells expressing the gut-homing receptors CCR9 and integrin α4β7 in HIV-infected individuals and found that many of these T cells remained in circulation rather than repopulating the small intestinal mucosa (170). This is likely because the expression of CCL25, the ligand of CCR9, was lower in the small intestine of HIV-infected individuals (170). The defective homing of CCR9^+^β7^+^CD4^+^ T cells, a T cell subset with a critical role in mucosal immune defense, into the gut of HIV-infected individuals correlated with high plasma concentrations of markers of mucosal damage, microbial translocation, and systemic T cell activation (170). These results suggest that alterations in CCR9^+^β7^+^CD4^+^ T cell homing to the gut prevented efficient mucosal immune reconstitution in HIV-infected individuals (170).

In 2019, Marelli-Berg et al. reported that the CCR9 receptor signals during naïve T cell priming and promotes the differentiation of α4β^+^7 IFN-γ-producing memory CD4^+^ T cell subset (171). These CCR9^+^β7^+^CD4^+^ T cells display a T_RM_ molecular signature, which is preferentially localized to the gastrointestinal tract and associated lymphoid tissue (171).

### 3.4 CCL25 chemokine immunoadjuvant to improve vaccines against infectious pathogens

Early in 2012, Kathuria et al. demonstrated the generation of antigen-specific T cells following systemic immunization with a DNA vaccine encoding CCL25 chemokine as immunoadjuvant (172).

In 2020, using a Chemokine-Adjuvanted Plasmid DNA expressing CCL25, Aldon et al. demonstrated in mice enhanced both splenic and intestinal, vaginal Ag-specific antibodies and T-cells primed by intramuscular immunization (173–176). This indicates that CCL25, a genetic chemokine immunoadjuvant, enhances vaccine Antigen-Specific humoral and cellular responses and induces homing to the gastrointestinal and female genital tract mucosae (173–176).

Similarly, in 2020, *Hsu et al.* demonstrated that parenteral administration of a virus-like particle-based vaccine formulated with CCL25 chemokine as an immunoadjuvant induced protective systemic and mucosal immune responses (144). This suggests that VLPs formulated with the CCL25 immunoadjuvant may serve as a potential vaccine strategy to protect against enteric viral infections.

More recently, in 2020, McKay et al. investigated the programming of T and B cells to home to the gastrointestinal and female genital tracts using genetic chemokine adjuvants (173). BALB/c mice were primed intramuscularly with plasmid DNA encoding a model Ag HIV-1 Env gp140 and selected chemokines/cytokines and boosted intravaginally with gp140 recombinant protein (173). CCL25 enhanced splenic and vaginal Ag-specific T cell responses, whereas CCL28 increased the levels of specific T cells only in the female genital tract (173). The levels of Ab were modulated in the systemic circulation, as well as the vaginal vault and intestinal lumen, with CCL20 playing a central role (173).

#### 3.4.1 Targeting the CCL25/CCR9 axis as therapies for inflammatory and infectious diseases

*CCL25/CCR9 chemokine axis and ulcerative colitis*: Ulcerative colitis patients display increased numbers of circulating pro-inflammatory monocyte human leukocyte antigen-DR [HLA-DR^hi^] monocytes expressing high levels of the gut-homing CCR9 receptor of CCL25 and tumor necrosis factor [TNF]- α. In 2016, Trivedi et al. demonstrated that intestinal CCL25 expression is increased in colitis and correlates with inflammatory activity (177, 178). CCL25 expression is upregulated in active colitis and correlates strongly with the burden of inflammatory bowel disease. Pathogenic CCR9^+^ T-cells undergo adhesion to stimulated hepatic endothelium more readily than CCR9^−^ T-cells. Accordingly, several clinical trials have been conducted to block the CCL25/CCR9 chemokine axis in the treatment of patients with various disease conditions (25, 179).

In 2017, Eberhardson et al. reported the results of a randomized, double-masked, placebo-controlled trial of CCR9-targeted leukapheresis treatment in patients with ulcerative colitis (179). Patients with ulcerative colitis were treated every second day with leukapheresis using either a CCL25 column or a placebo column for five sessions (179). This clinical trial aimed to evaluate the removal of circulating CCR9-expressing monocytes via leukapheresis in patients with moderate-to-severe ulcerative colitis, focusing on safety, tolerability, and immunological responses. Patients with ulcerative colitis were treated every second day with leukapheresis during five sessions with a CCL25 column or a placebo column. This clinical induction trial, utilizing CCL25-tailored leukapheresis, demonstrates the safe and effective removal of activated monocytes with a clinical effect in patients with ulcerative colitis (25, 179). Later, in 2018, Igaki et al. demonstrated MLN3126, an antagonist of the CCL25 chemokine receptor (122).

The receptor CCR9 and its ligand, CCL25, also play essential roles in gut inflammation and autoimmune colitis (133). Gut mucosa-homing DCs express CCR9 and are predominantly localized in the gut lining and thymus (133, 134). CCR9^+^ DCs are implicated in regulating gut inflammation and food allergyin the gut (133, 134). The differential interaction of CCR9^+^ DCs with T cells in secondary lymphoid tissues and mucosal sites provides crucial insights into immune regulation (133, 134). The phenotypes, distributions, and interactions of CCR9^+^ DCs have recently been reviewed by Pathak et al., who elucidate the functions and roles of CCR9+ DCs in inflammation (133, 134). In 2007, Saruta et al. demonstrated that CCR9^(+)^ T cells in Crohn’s disease are pro-inflammatory, supporting the rationale for using CCR9 antagonists in the treatment of human Crohn’s disease (136).

*CCL25/CCR9 chemokine axis and Inflammatory bowel disease*: The CCL25/CCR9 chemokine ligand-receptor pair has been reported to play a crucial role in small bowel immunity and inflammation (136). The CCR9/CCL25 axis contributes to the maintenance of mucosal T cell immunity and pathogenesis of inflammatory bowel disease (IBD) through the recruitment of pathogenic CCR9^+^ T cells into the gut mucosa (122). More recently, adoptive transfer of CCR9^(+)^ DCs in B6 mice promoted T_regs_ but reduced the Th17 cells in the GALT and suppressed the OVA-specific gut-allergic response; Pathak et al. suggested CCR9^(+)^ DCs have a regulatory function and may provide a new cellular therapeutic strategy to control gut inflammation and allergic immune reaction (133).

*CCL25/CCR9 chemokine axis and Crohn’s disease*: CCL25 is also necessary for the attraction and generation of B cells to the small intestine, lamina propria, and intraepithelial regions (180). Decreased circulating CCR9^+^CD4^+^ T helper cells are also associated with elevated levels of the. CCL25 chemokine in the salivary glands of patients with Sjögren’s syndrome, which facilitates their concerted migration (181). A randomized clinical trial using vercirnon, an oral CCR9 antagonist, versus placebo as induction therapy in active Crohn’s disease has been reported (182).

*CCL25/CCR9 chemokine axis and COVID-19 disease*: There is a consensus regarding the chemokine profile among COVID-19 patients, in which the variety of CXC and CC chemokines, as well as their association with COVID-19 vaccination, are major contributors to the immunopathology post-SARS-CoV-2 infection and remain important targets for therapy (42). The CC and CXC chemokines, in particular, appeared to contribute to the severity of COVID-19 disease (see **Table 1** and reference (42)). In late 2022, Khalid et al. sequenced pooled peripheral blood mononuclear cell (PBMC) transcriptomes from SARS-CoV-2 patients with moderate and critical clinical outcomes to identify novel host receptors and biomarkers that could inform the development of translational nanomedicines and vaccine therapies (183). In 2021, Su et al. provided evidence that the ORF7a protein induced the NF-κB-dictated production of pro-inflammatory cytokines and chemokines, including significant upregulation of CCL25 (among many other chemokines) in severely ill COVID-19 patients (184). The ORF7a protein is then proposed as a target to minimize uncontrolled inflammation in COVID-19 patients (184). The hyperproduction of chemokines in lung mucosal tissue significantly worsens the prognosis of COVID-19 disease. In 2022, Karimabad et al. reviewed the roles of CXC, CC, and C chemokines in the pathogenesis of COVID-19 and their use as surrogates of COVID-19 vaccine-induced innate and adaptive Immunity (42).

## 4 The mucosal chemokine (C-C motif) ligand 28 in human health and disease

The chemokine (C-C motif) ligand 28 (CCL28), is one of the most widely expressed mucosal CC chemokine, also known as mucosal-associated epithelial chemokine (MEC), CCK1 and SCYA28, discovered back in 2000 by Wang et al. (**Table 1**) (185). CCL28 is a dual homeostatic/inflammatory chemokine that plays a role in mucosal immunity, as a chemoattractant for immune cells expressing CCR10 (known as GPR2) and CCR3 receptors, and as a broad-spectrum antimicrobial protein (33, 125, 129, 185–192).

The human CCL28 gene is located on chromosome 5 at 5p12 (**Figure 1A**) and is transcribed into an RNA transcript of 373 nucleotides, comprising four exons (185, 192, 193). The gene encodes a 127-amino-acid CCL28 protein that includes a 22-amino-acid N-terminal signal peptide. The human CCL28 shares 76% nucleotide identity and 83% amino acid similarity with the mouse CCL28 (185, 192, 193). CCL28 is constitutively expressed in thymic dendritic cells and mucosal epithelial cells in the salivary glands, bronchial epithelium, and the digestive tract, and its expression is induced in the mammary glands. CCL28 is also expressed by columnar epithelial cells in the gut and lungs (**Figure 1B**).

In 2006 and 2007, Meurens et al. reported that, like mouse and human CCL28 and CCR10, the ovine CCL28 and CCR10 are expressed in porcine mucosal digestive tract tissues (127–129). The ovine CCL28 and CCR10 were cloned and sequenced, and their mRNA expression was subsequently assessed by q-PCR in several tissues, including thymus, gut-associated lymphoid tissue, and mammary gland, from young and adult sheep and in the fetal lamb during the development of the immune system (127–129). CCL28 mRNA was confirmed to be highly expressed in the large intestine, trachea, tonsils, and mammary glands, especially at the end of gestation (127–129). These results are consistent with previous observations in other species, suggesting similar roles for the CCL28 chemokine. In fetuses, mRNA for CCL28 and its receptor, CCR10, is expressed early in the thymus and mucosal tissues, including the small intestine and nasal mucosa (127–129). Furthermore, their expression increased towards the end of gestation. Consequently, CCL28 plays a vital role in the T cell colonization of fetal tissues, enabling the development of a functional immune system (127–129).

Knowledge of cellular and molecular immunology of the ocular mucosa-associated lymphoid tissue (OMIS) may aid in understanding ocular pathologies and in designing more effective immunization strategies to induce local anti-pathogen CD4^+^ and CD8^+^ T_RM_ cells, thereby protecting against ocular pathogens (58, 59). We have previously described OMIS and elucidated the structure and function of the humoral and cellular immune systems that protect the ocular mucosa (58, 59). A 2024 report by Dominguez-Lopez et al. showed that the gene expression of CCL28 was significantly upregulated in the conjunctiva of patients with dry eyes (57). CCL28 expression correlated positively with symptomatology, corneal staining, heat sensitivity threshold, and dendritic cell density (57). These results suggest that corneal and conjunctival epithelial cells could be a source of CCL28 on the ocular surface and that CCL28 might be involved in dry eye pathogenesis (57). Ocular mucosal immunoprophylactic and immunotherapeutic vaccine strategies have been evaluated to control the many pathogens that infect the ocular mucosa (58, 59). Topical ocular delivery of subunit adjuvanted vaccines against ocular herpes HSV-1 infection has been described (59, 194–197). Future challenges and issues related to the ocular mucosal delivery of molecularly defined subunit vaccines include how to generate efficient ocular mucosal CD4^+^ and CD8^+^ T_RM_ cells to combat invading ocular pathogens.

### 4.1 CCR10 and CCR3 are both C-C receptors of CCL28 chemokine

CCL28 is a β- or CC chemokine that is involved in host immunity through the interactions with its receptors CCR10 and CCR3. CCL28 chemokine is expressed by columnar epithelial cells in the gut, lung, breast, and salivary glands. One or both receptors are expressed by B and T cells, eosinophils, mast cells, and inflammatory cells, and drive their mucosal homing (33, 124, 125, 190, 198–201). The CCR10 Receptor, originally called orphan receptor GPR2, is a receptor for CCL28 (124, 125, 132, 190, 200, 202). CCR10 is most highly expressed in eosinophils and basophils but is also expressed in Th1 and Th2 cells and airway epithelial cells. The CCR10 receptor has been implicated in skin inflammation and is involved in the recruitment of regulatory T cells (T_regs_) into mucosal layers (188, 203–215). The CCR10/CCL28 axis influences the initiation and progression of mucosal inflammation in asthma by regulating the recruitment and retention of leukocyte populations in healthy and inflamed genital tracts.

The CCR receptor CCR3 is also a receptor for CCL28 that attracts B and T cells to mucosal tissues (26, 190, 216–226). Thus, CCR3 contributes to allergic reactions. CCR3 is also known as CD193 (26, 190, 219–226).

### 4.2 The CCL28/CCR10 chemokine ligand/receptor pair and the development of mucosal tissues-resident CD4^+^ and CD8^+^ T cells

The role of CCL28 chemokine in T cell trafficking and intestinal immunity was reported back in 2002 by Kunkel, Campbell, and Butcher (166). The CCL28 chemokine regulates the chemotaxis of cells that express the chemokine receptors CCR3 and CCR10. CCL28 is expressed by columnar epithelial cells in the gut, lung, breast, and salivary glands. It drives the mucosal homing of T cells expressing CCR10 and the migration of eosinophils expressing CCR3 (33, 125, 190). This chemokine is constitutively expressed in the colon. Still, its levels can be increased by pro-inflammatory cytokines and certain bacterial products, implying a role in effector cell recruitment to sites of epithelial injury (227–231). CCL28 has also been implicated in the migration of IgA-expressing cells to the mammary gland, salivary gland, intestine, and other mucosal tissues (227–231). It has also been shown to be a potential antimicrobial agent effective against specific pathogens, including Gram-negative and Gram-positive bacteria, as well as the fungus *Candida albicans* (227–231).

In humans, mice, and pigs, CCL28 plays a crucial role in the segregation and compartmentalization of the mucosal immune system by recruiting B and T cells expressing CCR10 to specific locations (125, 147, 188, 232–236), facilitating the migration of eosinophils expressing CCR3 (190, 237). CCL28 plays a crucial role in the migration of IgA-expressing cells to the mammary glands (238), salivary glands, intestines (123), and other mucosal tissues (33). In 2006, Eksteen et al. demonstrated that epithelial inflammation is associated with CCL28 production and the recruitment of regulatory T cells expressing CCR10. The authors suggested that CXCR3 promotes the recruitment of T_regs_ to inflamed tissues, and CCR10 allows them to respond to CCL28 secreted by epithelial cells, resulting in the accumulation of tissue-resident CCR10^+^ T_regs_ at mucosal surfaces. An indispensable role for CCL28 and its receptor, CCR10, in the accumulation of IgA antibody-secreting B cells in the lactating mammary gland was reported by Wilson and Butcher in 2004 (238, 239). In 2008, Morteau et al. reported an essential role for the chemokine receptor CCR10 in the accumulation of IgA antibody-secreting cells (239).

In 2022, Terefe et al. confirmed that CCR10 is involved in the trafficking, recruitment, and infiltration of T cells into epithelia, such as the skin, *through* its interactions with the chemokines CCL27 and CCL28 (232). Furthermore, CCL28 binds to the chemokine receptor CCR3. CCR3 and/or CCR10 receptors are expressed by T cells that are implicated in the pathogenesis of asthma.

Lazarus et al. demonstrate that the mucosae-associated CCL28 chemokine is selectively chemotactic for IgA-secreting B cells (ASC): CCL28 attracts IgA- but not IgG- or IgM-producing ASC from both intestinal and non-intestinal lymphoid and effector tissues, including the intestines, lungs, and lymph nodes draining the bronchopulmonary tree and oral cavity (33, 123–126). These findings suggest a broad and unifying role for CCL28 in the physiology of the mucosal IgA immune system (33, 123–126).

### 4.3 Role of CCL28/CCR10 axis in the development of CD4^+^ and CD8^+^ T_RM_ cells against genital herpes

**Figure 4:**
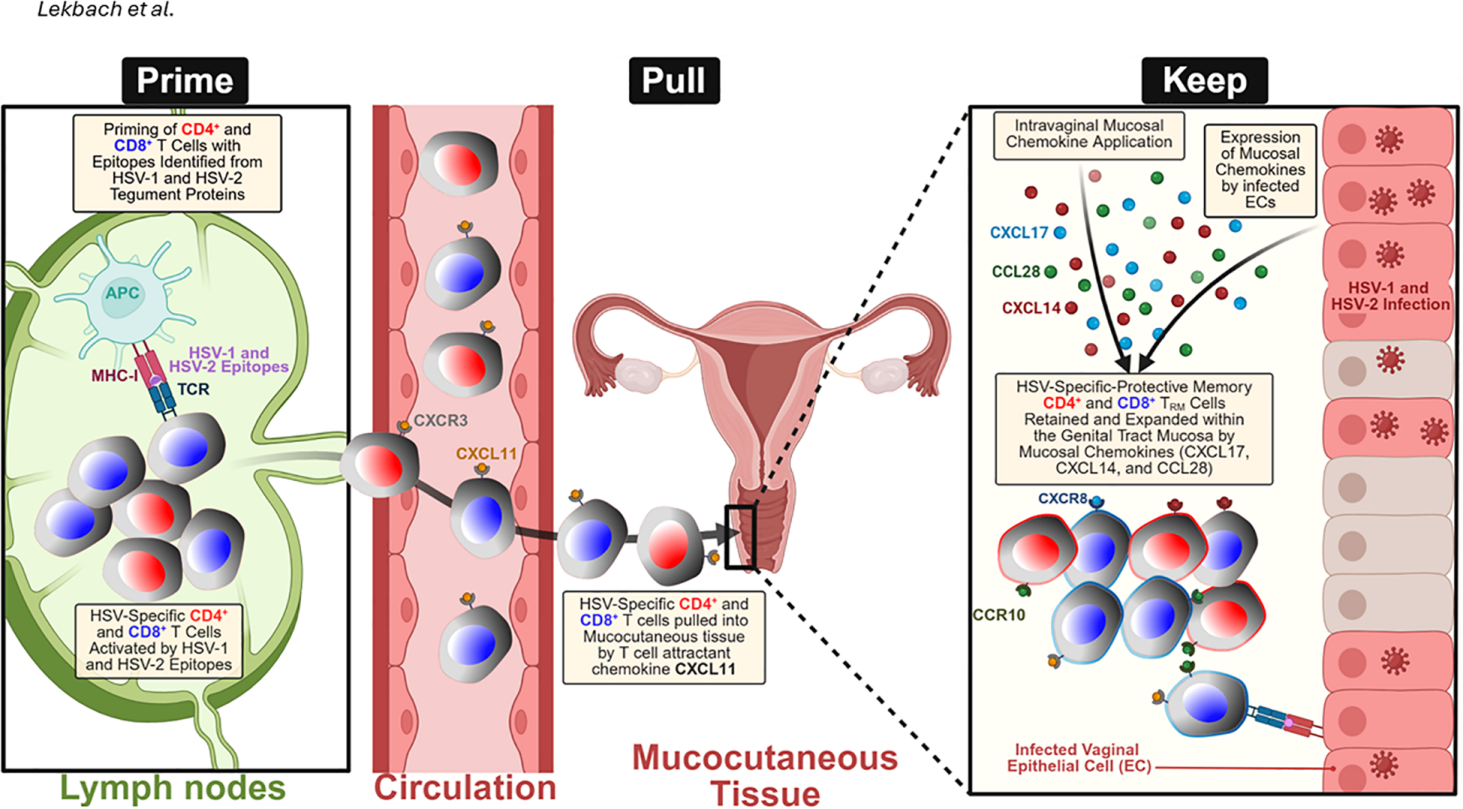
“Prime-Pull-Keep” (PPK) strategy for generating and maintaining HSV-2-specific tissue-resident memory T (TRM) cells in the mucocutaneous tissue: During the Prime phase, antigen-presenting cells (APCs) in the lymph nodes present HSV-1 and two tegument protein-derived epitopes to naïve CD4⁺ and CD8⁺ T cells, triggering their activation and expansion. During the Pull phase, activated HSV-specific T cells expressing CXCR3 are recruited from the circulation into the infected tissue by the T-cell-attracting chemokine CXCL11. During the Keep phase, the administration of mucosal chemokines, including CXCL14, CXCL17, and CCL28, alongside endogenous chemokine expression by HSV-1- or HSV-2-infected vaginal epithelial cells (VECs) and keratocytes, facilitates the retention and expansion of CD4⁺ and CD8⁺ T_RM_ cells within the genital tract. These effects are mediated through chemokine receptors, such as CCR10 and CXCR8, which promote long-term mucosal immune protection. Created with BioRender.com.

Indirect evidence in humans (105, 106) and direct experiments in animal models (102–104) suggest that successful control of herpes infection is associated with induction of robust and polyfunctional T_RM_ cells that reside within the vaginal sub-mucosal tissues (107). However, the female genital tract tissues appear to be immunologically restricted and remain “a closed immunological compartment, “ resistant to the homing of T cells that may originate from the draining lymph nodes and circulation (108, 109). Genital herpes infection of VECs likely triggered the production of inflammatory cytokines, such as IFN-gamma, TGF-beta, IL-1beta, and IL-6, which promote VECs: (1) to produce CCL28, and (2) increased CCR10 expression on local T cells contributing to homing and their retention of even more T_RM_ cells within the female genital tract tissues (**Figure 3**; **Table 2**) (108, 109). Moreover, following genital herpes infection, increased T-cell production, which attracts CXCL9 and CXCL10 chemokines, likely contributes to the recruitment of additional T_RM_ cells within the female genital tract tissues (**Figure 4**) (108, 109).

**Table 2:** Mucosal chemokines in T_RM_ cell biology.

Chemokine	Receptor	Expression in mucosal tissues	Stage of T_RM_ cell differentiation	Contribution to T_RM_ cell biology	References
CCL25	CCR9	Thymus, small intestine epithelium, and GALT mucosal epithelia	Priming/Homing stage of effector and memory T cells (e.g., in GALT-homing and imprinting).	Imprints GALT-homing (e.g., α4β7^+^CCR9^+^) of activated T cells and promotes differentiation of GALT-resident T_RM_-like cells. The CCL25-CCR9 ligand receptor interaction and signaling yields IFN^-^γ^+^ T_RM_ cells that preferentially reside in GALT.	PMID: 41012114, PMID: 30863398, PMID: 14592943, PMID: 38632596, PMID: 12393847, PMID: 39695803, PMID: 28207301 and PMID: 21300065
CCL28	CCR10 (and CCR3)	Salivary and mammary glands, respiratory tract (i.e., trachea, bronchus), colon, and female reproductive tract (i.e., uterus, cervix, vagina) mucosal epithelia	Recruitment and Retention of effector and memory T cells	Provides a recruitment and homeostatic chemotactic gradient for CCR10^+^ memory CD4^+^ and CD8^+^ T cells at mucosal epithelia. Enhances antiviral immunity by mobilizing CCR10^+^ effector memory CD8^+^ cells into the genital (vaginal) mucosa (protecting against HSV).	PMID: 41012114, PMID: 22684736, PMID: 38307549, PMID: 37222480, PMID: 36859428, PMID: 36155126, and PMID: 28207301, PMID: 33910988
CXCL17	CXCR8 (also known as GPR35)	Upper gastrointestinal tract (i.e., tongue, esophagus, stomach) and respiratory mucosa (i.e., trachea, lung) mucosal epithelia	Homing of effector and memory T cells	Recruits myeloid cells and memory T cells. CXCL17 attracts CXCR8^+^ T cells to mucosal tissues. Notably, CXCL17 drives CXCR8^+^CD8^+^ T_Eff/Mem_ and T_RM_ cells into the vaginal mucosa, enhancing protection against genital HSV in mice (via GPR35/CXCR8).	PMID: 41012114, PMID: 37210967, PMID: 33670758, PMID: 33515898, PMID: 29068046, PMID: 30860634, PMID: 28207301 and PMID: 29549178
CXCL14	No confirmed Receptor for CXCR4)	Skin, oral mucosa, gut (i.e., stomach and intestines), lung, kidney, and mammary gland mucosal epithelia	Homeostatic and Maintenance of effector and memory T cells	Has broad antimicrobial activity and by modulating innate cell recruitment and acting as a CXCR4 inhibitor, CXCL14 influences the niches that support T_RM_ survival and function in mucosal sites. High epithelial expression of CXCL14 sustains T_RM_ through tissue homeostasis and antimicrobial defense.	PMID: 41012114, PMID: 39931761, PMID: 39095323; PMID: 36012586, PMID: 31417179, PMID: 28382159, and PMID: 28207301

We recently performed bulk RNA sequencing of herpes-specific CD8^+^ T cells isolated from the peripheral blood mononuclear cells (PBMC) of women infected with HSV who were either symptomatic (SYMP) or asymptomatic (ASYMP) (83). Our analysis revealed distinct regulation of the chemokine pathway and significantly increased expression of the CC28 chemokine receptor, CCR10, in ASYMP compared with SYMP herpes-infected women. We further confirmed this result by flow cytometry analysis of immune cells from PBMCs of SYMP and ASYMP HSV-infected women (83). Thus, we detected increased CCR10 expression on HSV-specific CD8^+^ T cells in ASYMP compared with SYMP women, both at the transcriptional and translational levels (83). We also explored the role of the mucosal chemokine CCL28/CCR10 axis in protection against genital herpes infection and disease in mouse models (83). We used SYMP and ASYMP mice infected intravaginally with HSV. We found that an increased expression of CCL28 chemokine in the VM was associated with protection in ASYMP mice but not in SYMP mice. We further confirmed increased expression of the chemokine CCL28 in the VM of HSV-2-infected ASYMP mice using Western blotting and immunohistochemistry (83). Moreover, we found that the number of female genital tract CCR10^+^CD8^+^ T cells increased following intravaginal administration of the chemokine CXCL10 in HSV-2-infected mice (83). The corresponding increase in CCR10-expressing genital tract mucosal-resident memory T cells was demonstrated by flow cytometry, further suggesting a critical role for the mucosal chemokine CCL28/CCR10 axis in protective T-cell immunity against genital herpes (83).

The immune profile of cells in the VM of infected mice showed that CCL28^(-/-)^ mice had fewer CCR10-expressing CD8^+^ and CD4^+^ T cells, as well as a lower frequency of CCR10^+^CD44^+^ memory CD8^+^ T cells, compared with WT mice (83). The role of the CCL28/CCR10 chemokine axis in the mobilization of IgA-secreting cells in the mucosa is well established in the literature (83). To further elucidate the role of CCL28 and its receptor in humoral immunity during genital herpes infection, we investigated CCR10 expression on B cells in the VM. Interestingly, most memory B cells in the VM of these mice expressed the chemokine receptor CCR10. There was also a decrease in the frequency of CD27^+^B220^+^ memory B cells in these CCL28^(-/-)^ mice (83). The increased frequency of CCR10-expressing CD8^+^ T cells in asymptomatic individuals with herpes may suggest an association between the mucosal chemokine CCL28 and protection against herpes infection. Thus, the mucosal chemokine CCL28 mediates protection from disease severity by mobilizing both CCR10^+^CD44^+^ memory CD8^+^ T cells and CCR10^+^B220^+^CD27^+^ memory B cells to the VM (83) (**Table 2**).

We recently generated novel knockout (KO) mice for the CCL28 chemokine and the CCR10 receptor and are currently studying their phenotypes upon infection with virulent and non-virulent strains of HSV-1 and HSV-2.

### 4.4 Targeting the CCL28/CCR10 chemokine/receptor axis to improve vaccines and immunotherapies against herpetic diseases

During the last 20 years, only a single vaccine strategy (adjuvanted recombinant HSV glycoprotein D (gD), with or without gB) has been tested and retested in clinical trials (91). Despite inducing potent HSV-specific neutralizing antibodies, this strategy failed to meet the primary endpoint of reducing herpes disease (240). These failures emphasize the need to induce T-cell-mediated immunity (241). Following the resolution of viral infections, a long-lived memory CD8^+^ T cell subset that protects against secondary (2°) infections is generated (242–246). This memory CD8^+^ T cell subset is heterogeneous but can be divided into three major subsets: (1) effector memory CD8^+^ T cells (CD8^+^ T_EM_ cells); (2) central memory CD8^+^ T cells (CD8^+^ T_CM_ cells); and (3) CD8^+^ T cells (CD8^+^ T_RM_ cells) (108). The three significant memory subpopulations of T cells differ in their phenotypes, functions, and anatomical distributions. T_CM_ cells are CD62L^high^CCR7^high^CD103^low^. T_EM_ cells are CD62L^low^CCR7^low^CD103^low^. T_RM_ cells are CD62L^low^CCR7^low^CD103^high^CD11a^high^CD69^high^ (108, 247, 248). CD8^+^ T_RM_ cells are found in the female genital tract and offer protection in mouse models of genital herpes (113). CD8^+^ T_EM_ cells are also found in the dermal-epidermal junction in the female genital tract (32, 115). Once formed, T_RM_ cells do not re-enter the circulation and play an essential role in locally guarding mucosal tissues against secondary (2°) infections. However, the precise mechanisms by which non-circulating mucosa-resident memory CD8^+^ T_RM_ cells are formed, maintained, and expanded remain incompletely understood. In a recent study, we found that a high frequency of CD8^+^ T_RM_ cells is retained in the female genital tract of HSV-infected asymptomatic mice compared to symptomatic mice and that this is associated with CCL28 mucosal chemokine production. Specifically, we demonstrated that higher frequencies of CCL28-dependent antiviral CD8^+^ T_RM_ cells in the female genital tract are a key mediator of protection against genital herpes, consistent with previous reports (90, 103, 110–112, 115, 249). Since the primary cell target of HSV-2 is VECs, the key to achieving anti-herpes mucosal immunity is likely to boost the frequencies of HSV-specific CD8^+^ T_RM_ cells in the female genital tract that can expand locally and persist in the long term. CD8^+^ T_RM_ cells persist long-term in tissues and are often located at the epithelial borders of mucosal tissues (29, 250–253). However, little information exists on the mechanisms regulating the formation, retention, and expansion of vaginal-mucosa-resident CD8^+^ T_RM_ cells. This report is the first to show that CCL28/CCR10 chemokine axis-mediated signals may be required for high frequencies of female genital tract antiviral CD8^+^ T_RM_ cells. It remains to determine the mechanism of expansion and long-term retention of these CD8^+^ T_RM_ cells within the female genital tract. Such knowledge would inform the design of innovative vaccines that induce CD8^+^ T_RM_ cell-mediated protection against genital herpes. Collectively, this knowledge could significantly enhance our understanding of mucosal immunity and provide a unique opportunity to develop a robust, long-lasting genital herpes vaccine, with a substantial impact on the epidemiology of this disease. To our knowledge, our study represents the first in-depth analysis of the role of the CCL28/CCR10 chemokine axis in anti-herpes T- and B-cell responses in the VM during HSV-2 infection (83) (**Table 2**). We demonstrated that following intravaginal HSV-2 re-infection of B6 mice, high production of the CCL28 chemokine in the VM was associated with increased infiltration of CCR10^+^CD44^+^ memory CD8^+^ T cells and CD27^+^B220^+^ memory B cells in the VM (83) (**Table 2**). Our findings could further aid in immunotherapeutic approaches for genital herpes. In 2020, Aldon et al. demonstrated that the use of Chemokine-Adjuvanted Plasmid DNA expressing CCL28 increased the levels of specific T cells only in the female genital tract (173–176). This indicates that CCL28, a genetic chemokine immunoadjuvant, enhances vaccine Antigen-Specific humoral and cellular responses and induces homing to the female genital mucosa (173–176).

In 2021, Hu et al. demonstrated that CCL28 facilitated HSV-2 gD-induced protective systemic immunity against genital herpes (233). They showed that CCL28 enhanced robust antibody responses to the HSV-2 gD ectodomain (gD-306aa) and to Th1- and Th2-like responses that were recalled after the HSV-2 challenge. Interestingly, as expected, the mucosal chemokine CCL28 appeared to be more effective than CCL19 in promoting gD-specific immune responses and in promoting T-cell migration to secondary lymphoid tissues. Notably, both CCL19 and CCL28 significantly facilitated gD-induced protective mucosal immune responses in the genital tract. The findings collectively highlight the potential of the mucosal chemokine CCL28, in combination with gD, as a strategy for controlling HSV-2 infection. Recently, Huppler et al. demonstrated that CCL28 is also a potent therapeutic agent for *Oropharyngeal Candidiasis* (254).

In 2022, Terefe et al. confirmed that CCR10 is implicated in the trafficking, recruitment, and infiltration of T cells into epithelia *via* ligation by the chemokines CCL27 and CCL28 (232).

The above reports support the use of mucosal chemokines as an immune adjuvant to augment parenteral subunit vaccine-induced B- and T-cell immunity at mucosal surfaces (**Table 2**).

## 5 The mucosal chemokine (C-X-C motif) ligand 14 in human health and disease

The mucosal chemokine CXCL14 is a small cytokine belonging to the CXC chemokine family known as breast and kidney-expressed chemokine (BRAK chemokines), as reported back in 1999 by Hromas et al. (**Table 1**) (30, 255). The gene for CXCL14 contains four exons and is located on chromosome 5q31.1 in humans (**Figure 1A**) (30, 255). Mature CXCL14 has many of the conserved features of the CXC chemokine subfamily but has some differences, such as a shorter N-terminus and five extra amino acids in the region between its third and fourth cysteines (30, 255). Between 2006 and 2008, Meuter and Moser described the constitutive expression of CXCL14 in healthy human and murine epithelial tissues (31, 256, 257). CXCL14 is constitutively expressed in several normal tissues, including the brain, breast, cervix, lung, kidney, and skin, where its cellular source is thought to be fibroblasts (**Figure 1B**) (258).

CXCL14, a relatively novel chemokine, is a non-ELR (glutamic acid-leucine-arginine) chemokine with a broad spectrum of biological activities. Squamous epithelia, in particular, express high levels of CXCL14, supporting the notion that CXCL14 plays a homeostatic role in the skin. CXCL14 chemokine is chemotactic for monocytes in the presence of prostaglandin E2 (PGE2), an inflammatory mediator (258). It is also a potent chemoattractant and activator of dendritic cells (259–261) and activated natural killer (NK) cells (262–264). CXCL14 also inhibits angiogenesis, possibly by blocking endothelial cell chemotaxis (262).

A 2024 report by Dominguez-Lopez et al. showed that CXCL14 gene expression was significantly upregulated in conjunctival samples from patients with dry eyes (57). CXCL14 expression correlated positively with age, ocular pain, conjunctival staining, tactile sensitivity, and image reflectivity (57). These results suggest that corneal and conjunctival epithelial cells could be a source of CXCL14 on the ocular surface (57). CXCL14 has an atypical, yet highly conserved, primary structure characterized by a short N-terminus and high sequence identity between human and mouse. Proinflammatory chemokine CXCL14 activates MAS-related G protein-coupled receptor MRGPRX2 and its putative mouse ortholog MRGPRB2.

There is no widely accepted specific receptor for CXCL14 (265–273). One study predicted potential receptors using a protein interaction network database (STRING), suggesting that CXCR4, CXCR3, CXCR2, CCR2, CCR1, CXCR5, CCR7, CXCR1, and CCR5 may interact closely with CXCL14 and could be potential receptors (271, 274). Other studies suggested that ACKR2, CXCR4, GPR25, and GPR182 (271, 274, 275) are potential receptors for CXCL14.

### 5.1 Role of CXCL14 chemokine axis in the development of CD4^+^ and CD8^+^ T_RM_ cells

Although CXCL14 induces chemotaxis of monocytic cells at high concentrations, its physiological role in leukocyte trafficking remains elusive (276, 277). CXCL14 primarily regulates T cell migration but also plays a distinct role in antimicrobial immunity and is proposed to combat bacteria at the earliest stage of infection, well before the establishment of inflammation (31).

In 2016, Pyeon et al. demonstrated the protective role of the CXCL14 chemokine in preventing human papillomavirus (HPV) infection, which can lead to cancer, through a mechanism that induces NK, CD4^+,^and CD8^+^ T cells (278–283). Conversely, immune evasion is demonstrated by HPVs, which suppress antitumor immune responses through epigenetic downregulation of CXCL14 expression (282). Pyeon et al. then analyzed gene-expression changes across all known chemokines and their receptors using our global gene-expression datasets from human HPV-positive and -negative head and neck cancer and cervical tissue specimens at different disease stages (282). While many proinflammatory chemokines are upregulated throughout cancer progression, CXCL14 is markedly downregulated in HPV-positive cancers in humans (282). Restoration of CXCL14 expression in HPV-positive mouse oropharyngeal carcinoma cells clears tumors in immunocompetent syngeneic mice but not in Rag1-deficient mice (282).

Furthermore, CXCL14 re-expression significantly increases the infiltration of natural killer (NK) cells, CD4^+ T cells^, and CD8^+^ T cells into the tumor-draining lymph nodes *in vivo* (282). *In vitro* transwell migration assays show that CXCL14 re-expression induces chemotaxis of NK, CD4^+^, and CD8^+^ T cells (282). Later, in 2019, the Pyeon et al. group demonstrated that CXCL14 suppresses human papillomavirus-associated head and neck cancer through antigen-specific CD8^+^ T-cell responses by upregulating MHC-I expression (279, 280). In 2024, based on the above strong results, Pyeon et al. proposed targeting the CXCL14 chemokine signaling pathway as a practical approach for cancer immunotherapy to halt HPV-cancer progression (278).

Given its roles in NK, CD4^+^, and CD8^+^ T-cell homing to tissues, CXCL14 stands out as a promising candidate for novel cell immunotherapies (**Figure 3**). However, it remains to be determined: the molecular mechanism that regulates CXCL14-mediated activity; how to deliver CXCL14; the appropriate dose; and which combinations with existing approved therapies may enhance NK, CD4^+^, and CD8^+^ T cell responses. We recently generated knockout (KO) mice for the CXCL14 chemokine and are currently studying their phenotype upon infection with HSV-1 and HSV-2. Preliminary, unpublished data suggest that CXCL14 plays a significant role in the homing of CD4+ and CD8+ T cells to peripheral tissues. In CXCL14^(⁻/⁻)^ mice intravaginally infected with HSV-2, we observed increased disease severity and mortality, accompanied by reduced infiltration of CD4^+^ and CD8^+^ T cells at the site of infection and elevated viral loads.

In addition to CXCL14, in 2023-2024, Han et al. reported that CXCL13, primarily produced by CD4^+^ T cells, is an important chemokine involved in the recruitment of CXCR5-expressing naïve B and T follicular helper cells to the lungs ( (284) and Abstract AAI, 2024).

In summary, the initial differentiation of T_RM_ cells is primarily driven by local tissue signals, including cytokines such as TGF-β, IL-15, and IL-33, as well as transcription factors like Hobit and BLIMP-1 (285, 286). The chemokines listed here do not primarily generate the T_RM_ lineage itself but create the necessary microenvironment for their homing and subsequent survival/retention. (285, 286). (**1**) Homing: CCL25 and CCL28 act as crucial “pull” signals, expressed constitutively by epithelial cells, guiding circulating T cell precursors to specific mucosal sites (e.g., small intestine for CCL25) where they can differentiate into T_RM_ cells (285, 286). CXCL17 also functions as a chemoattractant for T cells and antigen-presenting cells (APCs) to mucosal tissues. (**2**) Maintenance: Once established, these chemokines, along with adhesion molecules such as CD103 (which binds to E-cadherin) and CD69 (which prevents S1P-mediated egress), contribute to the long-term residency and survival of T_RM_ cells in their respective niches (285, 286). The continuous presence of the chemokines acts as a “keep” signal, retaining cells locally. (**3**) Reactivation: Upon local antigen re-encounter, pre-existing T_RM_ cells are rapidly reactivated (e.g., secreting IFN-γ \ and TNF-α) to control infection and recruit circulating immune cells, but the specific role of these four chemokines in the reactivation process itself is less defined compared to their roles in homing and maintenance (285). The expression profile during inflammation may change (e.g., elevated CCL28/CXCL17 in dry eye or infection), but their primary role remains homing/retention (285, 286).

## 6 The prime/pull/keep immunotherapy for genital herpes

Despite decades of effort, an effective vaccine or immunotherapy for genital herpes remains elusive. Traditional approaches, including inactivated “killed” virus, live-attenuated, replication-defective, and subunit glycoprotein vaccines, have largely failed to prevent recurrent HSV-2 disease (287). For example, a glycoprotein D subunit vaccine with adjuvant (aluminum salt) induced neutralizing antibodies but provided only modest and inconsistent protection in clinical trials. These challenges underscore the need for a new strategy that elicits robust T-cell immunity, rather than relying solely on antibodies. Researchers have observed that individuals with better control of genital herpes have robust HSV-2-specific CD4^+^ and CD8^+^ T cells residing in the vaginal mucosa (VM, the site of recurrent lesions) and their dorsal root ganglia (DRG, the site of viral latency) (288). An ideal therapeutic vaccine should therefore induce such T_RM_ cells at both the central neuronal immunity in DRG and the peripheral epithelial immunity in VM. However, memory T cells do not circulate freely into immune-restrictive sites, such as the VM, skin, or lung airways, at steady state; instead, their trafficking is governed by chemokine gradients induced by local infection or inflammation (52). Chemokines such as CXCL9 and CXCL10, which act through CXCR3, and mucosal chemokines such as CXCL17, CXCL14, and CCL28 have been shown to recruit and retain functional memory T cells within mucosal tissues (289). Building on this principle, the Prime/Pull/Keep (PPK) strategy represents this paradigm shift: first, prime the host with key HSV antigens to generate a broad T-cell response, then pull those T cells into infected tissues using chemokine cues, and finally keep them on site for long-term protection (**Figure 4**). In HSV-2-infected guinea pigs, we demonstrated that intravaginal administration of a neurotropic AAV8 vector encoding CXCL11 significantly enhanced the infiltration of functional CXCR3^+^ CD4^+^ and CD8^+^ T_RM_ and T_EM_ cells in both the DRG and VM, resulting in a substantial reduction in viral shedding and lesion recurrence (290). Further incorporation of mucosal chemokines, such as CCL28 and CXCL17, has demonstrated additive benefits, facilitating the long-term retention of CCR10^+^ and CXCR8^+^ memory T cells within the VM (4, 83) (**Table 3**). By establishing sustained antiviral T-cell immunity in both central and peripheral target tissues, the PPK strategy represents a promising paradigm shift in the development of effective immunotherapeutics against genital herpes. We used Guinea pigs because consensus among herpes scientists is that the guinea pig model, which develops spontaneous viral reactivation and human-like recurrent genital herpes disease, is the gold standard and a widely used small-animal model for preclinical testing of vaccine candidates translatable to humans (99, 290–300).

**Table 3:** Role of mucosal chemokine in HSV-1/2 infections.

Chemokine	Genital mucosa findings (HSV-1/2)	Ocular mucosa findings (HSV-1/2)	Key reference (2022–2025)
CCL25	Primarily a gut-associated chemokine (CCR9 ligand) expressed in small intestinal and thymic epithelium. No significant findings in genital HSV – it is not known to be induced or required in vaginal HSV infection (mucosal immunity to HSV-2 does not appear to involve CCL25).	No data in ocular HSV. (CCL25 is tissue-specific to the gastrointestinal tract; it is not typically expressed in ocular mucosa)	Meurens et al., 2006 – *J. Interferon Cytokine Res.* (CCL25 in mucosal immunity context); Smith et al., 2022 – *Front. Immunol.* (notes major mucosal chemokines and need for further study in HSV).
CCL28	Protective role: Homeostatically produced in vaginal mucosa. Elevated in asymptomatic HSV-infected individuals and mice, correlating with higher HSV-specific effector memory CCR10^+^ CD8^+^ T cells and memory B cells in vaginal tissue. CCL28-CCR10 axis mobilizes antiviral lymphocytes to infection site; CCL28knockout mice have increased susceptibility to genital HSV-2 and reduced mucosal memory T/B cell infiltration.	Not studied in ocular HSV. (Ocular surface epithelial cells can secrete CCL28 during inflammation, but its role in HSV-1 ocular infection is undetermined. Excess chemokine in eye may contribute to immunopathology rather than protection.)	Dhanushkodi et al., 2023 – *J. Immunol.* 211(1):118–129 (demonstrates CCL28’s role in recruiting CCR10^+^ T and B cells for genital HSV protection; DOI: 10.4049/jimmunol.2300093).
CXCL17	Protective role: Constitutively present in vaginal mucosa and induced by HSV. Higher CXCL17 levels observed in protected mice after intravaginal HSV exposure, accompanied by increased CXCR8^+^ CD8^+^ effector memory and CD103^+ tissue-resident T cells in the vaginal mucosa. In CXCL17 knockout mice, genital HSV-1 infection leads to fewer tissue CD8 T cells, more viral replication, and more latency in ganglia, indicating CXCL17 is crucial for mobilizing protective CD8^+^ T cells to the genital mucosa.	Not studied in ocular HSV. (No published evidence of CXCL17’s role in HSV-1 eye infections. Ocular herpes lesions are driven by an intense chemokine/cytokine storm causing inflammation, so any CXCL17 effect is uncharacterized. It has been suggested as a mucosal immunomodulator, but no direct data in the eye.)	Srivastava et al., 2018 – *J. Immunol.* 200(8):2915–2926 (CXCL17 mediates recruitment of CXCR8^+^ CD8 T_EM_ and T_RM_, protecting against vaginal HSV; DOI: 10.4049/jimmunol.1701474). Smith et al., 2022 – *Front. Immunol.* (review, notes chemokine CXCL17 in HSV mucosal immunity).
CXCL14	Constitutively expressed in mucosal epithelium; specific role in genital HSV infection remains unclear (no direct studies). Proposed to participate in basal immune surveillance but not confirmed in HSV-2 models.	Upregulated on ocular surface under inflammatory stress; no HSV-specific data. Ocular infection triggers broad chemokine release that mainly contributes to tissue damage rather than protection.	Dhanushkodi et al., 2023 – J. Immunol. (211(1):118–129, discusses CXCL14 as a mucosal chemokine; DOI: 10.4049/jimmunol.2300093).

## 7 Targeted PPK immunotherapy for ocular herpes

**Figure 5:**
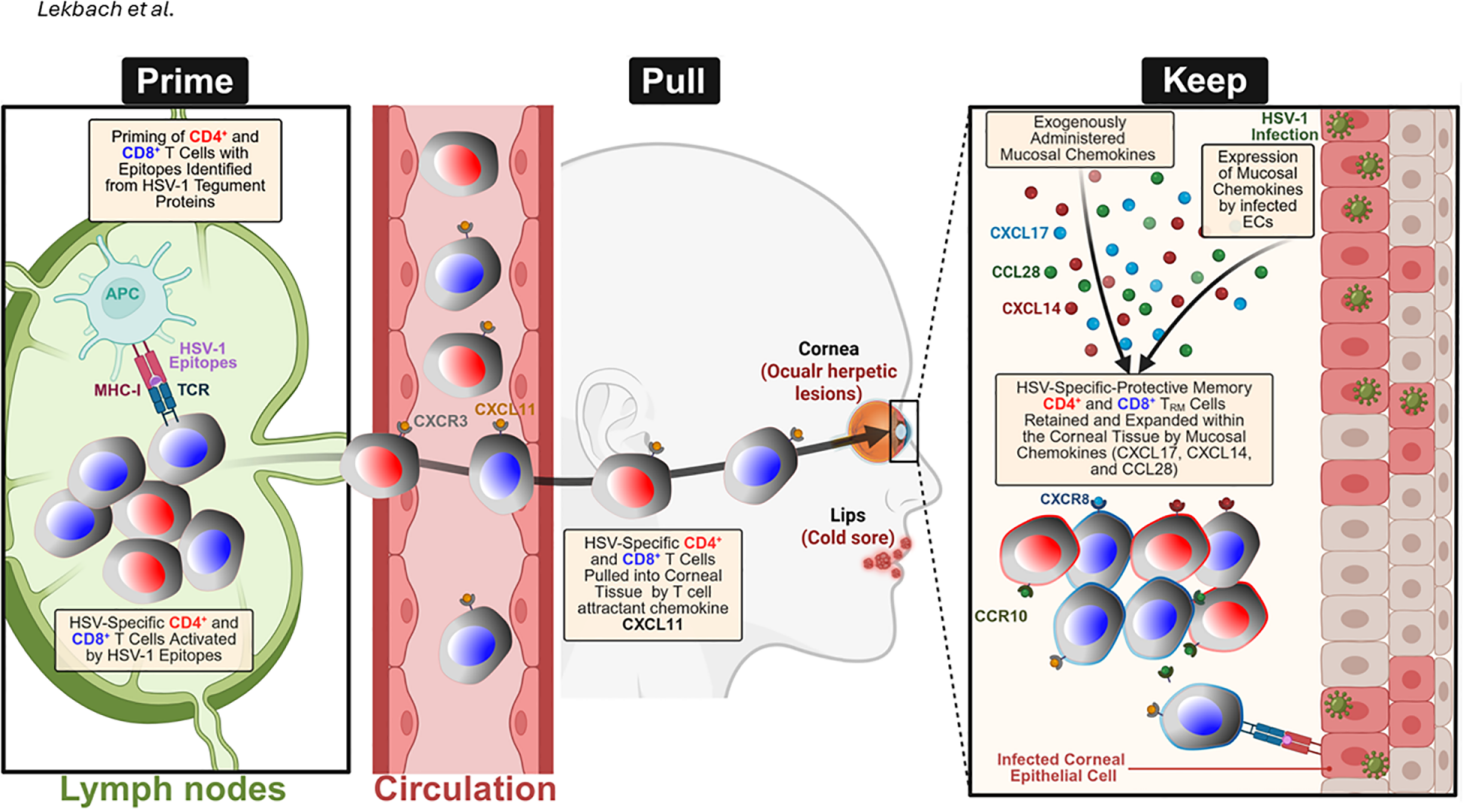
Distinct phases of the “Prime-Pull-Keep” (PPK) approach facilitate the generation and maintenance of HSV-1-specific T cell receptor (TRM) cells in the corneal mucosa: Initially, in the Prime phase, naïve CD4⁺ and CD8⁺ T cells are activated in regional lymph nodes upon recognition of HSV-1 antigens presented by antigen-presenting cells (APCs). The Pull phase involves CXCL11-mediated recruitment of circulating HSV-1-specific T cells, which express CXCR3, into the infected corneal tissue. During the Keep phase, chemokines such as CXCL14, CXCL17, and CCL28, produced locally by infected corneal epithelial cells or administered exogenously, enhance the persistence and expansion of TRM cells in the cornea. This retention is directed by chemokine receptors, including CXCR8 and CCR10, which support a robust and localized antiviral immune defense. Created with BioRender.com.

HSV-1 establishes lifelong latency in the trigeminal ganglion (TG) and is responsible for recurrent ocular herpes (301). Current antiviral therapies offer only temporary suppression, and vaccine efforts, such as adjuvanted glycoprotein subunit vaccines, have failed to prevent recurrence, highlighting the need for innovative strategies that elicit local antiviral immunity (91). The PPK approach represents a targeted immunotherapeutic solution designed to restore and sustain T_RM_ cells at sites of latency and reactivation. Recently, we tested a novel PPK therapeutic vaccine in an HLA-A*0201 transgenic rabbit model of ocular herpes (302). The vaccine combined HSV-1 CD8^+^ and CD4^+^ T-cell epitopes (prime) with the chemokine CXCL11 (pull) to recruit antiviral T cells into the TG, and IL-2/IL-15 (keep) to support their local maintenance. This strategy significantly increased TG-infiltrating CD8^+^ T cells and reduced both corneal disease and viral shedding, highlighting the potential of tissue-targeted immunotherapy to control HSV-1 reactivation. Moreover, mucosal chemokines such as CCL28, CXCL14, and CXCL17 are expressed in the cornea under homeostatic conditions (57). They could be incorporated into the PPK strategy to help retain antiviral memory T cells within infected ocular tissues, reinforcing local immune surveillance (**Figure 5**). By directing and retaining effector and memory T cells precisely at sites of HSV-1 persistence, the PPK strategy offers a promising path toward durable control of ocular herpes reactivation.
